# Environmental Impact on Vascular Development Predicted by High-Throughput Screening

**DOI:** 10.1289/ehp.1103412

**Published:** 2011-07-25

**Authors:** Nicole C. Kleinstreuer, Richard S. Judson, David M. Reif, Nisha S. Sipes, Amar V. Singh, Kelly J. Chandler, Rob DeWoskin, David J. Dix, Robert J. Kavlock, Thomas B. Knudsen

**Affiliations:** 1 National Center for Computational Toxiciology, Office of Research and Development, U.S. Environmental Protection Agency, Research Triangle Park, North Carolina, USA; 2 Lockheed Martin, Research Triangle Park, North Carolina, USA; 3 National Health and Environmental Effects Research Laboratory, and; 4 National Center for Environmental Assessment, Office of Research and Development, U.S. Environmental Protection Agency, Research Triangle Park, North Carolina, USA

**Keywords:** angiogenesis, developmental toxicity, high-throughput screening (HTS), thalidomide, vascular development

## Abstract

Background: Understanding health risks to embryonic development from exposure to environmental chemicals is a significant challenge given the diverse chemical landscape and paucity of data for most of these compounds. High-throughput screening (HTS) in the U.S. Environmental Protection Agency (EPA) ToxCast™ project provides vast data on an expanding chemical library currently consisting of > 1,000 unique compounds across > 500 *in vitro* assays in phase I (complete) and Phase II (under way). This public data set can be used to evaluate concentration-dependent effects on many diverse biological targets and build predictive models of prototypical toxicity pathways that can aid decision making for assessments of human developmental health and disease.

Objective: We mined the ToxCast phase I data set to identify signatures for potential chemical disruption of blood vessel formation and remodeling.

Methods: ToxCast phase I screened 309 chemicals using 467 HTS assays across nine assay technology platforms. The assays measured direct interactions between chemicals and molecular targets (receptors, enzymes), as well as downstream effects on reporter gene activity or cellular consequences. We ranked the chemicals according to individual vascular bioactivity score and visualized the ranking using ToxPi (Toxicological Priority Index) profiles.

Results: Targets in inflammatory chemokine signaling, the vascular endothelial growth factor pathway, and the plasminogen-activating system were strongly perturbed by some chemicals, and we found positive correlations with developmental effects from the U.S. EPA ToxRefDB (Toxicological Reference Database) *in vivo* database containing prenatal rat and rabbit guideline studies. We observed distinctly different correlative patterns for chemicals with effects in rabbits versus rats, despite derivation of *in vitro* signatures based on human cells and cell-free biochemical targets, implying conservation but potentially differential contributions of developmental pathways among species. Follow-up analysis with antiangiogenic thalidomide analogs and additional *in vitro* vascular targets showed *in vitro* activity consistent with the most active environmental chemicals tested here.

Conclusions: We predicted that blood vessel development is a target for environmental chemicals acting as putative vascular disruptor compounds (pVDCs) and identified potential species differences in sensitive vascular developmental pathways.

We currently face significant challenges in understanding developmental health risks associated with the thousands of diverse compounds entering the environment (Martin et al. 2009b). Traditional prenatal animal testing is a resource-intensive, low-throughput approach that yields limited mechanistic information about biological pathways and potential adverse consequences in humans, motivating a new paradigm for toxicity evaluation (National Research Council 2007). To this end, the U.S. Environmental Protection Agency (EPA) ToxCast™ research project (Dix et al. 2007) and the federal Tox21 consortium (Collins et al. 2008) have initiated a large-scale effort to profile the biological activities of a large number of chemicals across multiple *in vitro* assays, largely focused on human cellular and molecular targets, and to compile this information into predictive models of toxicity. Combined approaches using high-throughput screening (HTS) and high-content screening (HCS) platforms with computational (*in silico*) systems modeling efforts aim to identify sensitive molecular targets of chemicals, the biological pathways relevant to these targets, and their integration into modes of human developmental toxicity. Analysis of the ToxCast data set has revealed significant activity across a number of biological targets known to participate in blood vessel formation, maintenance, and remodeling. Examining perturbations in these crucial processes constitutes a focus for predictive modeling and chemical prioritization, and this proof-of-concept study on a prototypical toxicity pathway—vascular development—demonstrates the opportunity to increase mechanistic understanding of prenatal health and disease.

The cardiovascular system is the first organ system that develops to a functional state in the vertebrate embryo, reflecting the critical need for nutrient delivery and waste removal (Ferrara 2004). Blood vessel development occurs by two successive processes: vasculogenesis, *in situ* formation of nascent vessels from angioblasts leading to a primary capillary plexus, and expansion of this plexus by subsequent pruning and reorganization of endothelial cells through angiogenesis (Czirok et al. 2008). Disruption of vascular development has been directly correlated with prenatal loss, malformations, maternal placental complications, and neurodevelopmental problems (D’Amato et al. 1994; Ema et al. 2010; Hanson and Gottesman 2005; Hoyme et al. 1990; Klauber et al. 1997; Tideman et al. 2007). HTS of a 200-chemical small-molecule library in transgenic zebrafish embryos has revealed site-specific disruption of vascular development by several mechanistically diverse compounds, such as angiotensin-converting enzyme inhibitors, microtubule inhibitors, and estrogenic mycotoxins (Kitambi et al. 2009). Thalidomide is perhaps the best-known example of a vascular-disruptive developmental toxicant. Originally prescribed as an antinausea agent in the 1960s, the developmental toxicity of the drug was discovered after thousands of tragic cases of skeletal appendicular malformations, microphthalmia, and fetal loss occurred in humans (Mellin and Katzenstein 1962). It has since been shown to inhibit angiogenesis (D’Amato et al. 1994) via prevention of filopodial extensions from the endothelial tip cell in immature embryonic blood vessels (Therapontos et al. 2009), leading to secondary effects on gene expression, generation of reactive oxygen species, and cell death in neighboring tissue (Ito et al. 2010; Vargesson 2009).

When analyzing the HTS-HCS data, we strive to address several important questions. Do environmental chemicals that disrupt blood vessel development also cause developmental toxicity? For a subset of these chemicals, is the disruption of blood vessel development necessary and sufficient to account for the developmental toxicity? Given the apical, nonspecific nature of the observed end points *in vivo* (e.g., decreased fetal body weight, delayed skeletal ossification), which are typical of regulatory guideline developmental toxicity studies in rats and rabbits, more detailed information is required to determine whether vascular disruption is the true mechanism by which a chemical acts. The ToxCast data, covering a wide variety of highly specific mechanistic end points, strives to meet this need and provide a more in-depth description of potential chemical toxicity.

In light of the global importance of blood vessel development in cancer, developmental toxicity, and reproductive effects of pharmaceutical compounds, in the present study we examined whether putative vascular disruptor compounds (pVDCs) can be used as a class predictor for environmental chemicals. Here, we define pVDCs to include compounds that may directly perturb processes fundamental to vascular development. The mode-of-action processes could include vascular patterning–remodeling or utero-placental circulation, with specificity that may vary depending on mechanisms intrinsic to the embryo, placenta, or mother.

## Materials and Methods

*Source data.* Phase I of ToxCast (U.S. EPA 2010a) employed a chemical library of 309 unique structures, most of which are registered food-use pesticides with extensive animal bioassay data available. Chemicals were screened using 467 HTS assays across nine assay technology platforms with active chemicals run in concentration-dependent dose–response format (Judson et al. 2010). Assays measured direct interactions between chemicals and molecular targets (receptors, enzymes), as well as downstream effects on reporter gene activity or cellular consequences. For each assay–chemical combination, using automated curve fitting, we derived either an AC_50_ (half-maximal activity concentration) or LEC (lowest effective concentration, statistically significant change from controls), where a default value of 1 M was assigned for inactive chemicals. Pathway, process, and disease-based perturbation scores were constructed by mapping assays to genes and then to pathways from Gene Ontology (GO 2010), Kyoto Encyclopedia of Genes and Genomes (KEGG; Kanehisa Laboratories 2010), Ingenuity Pathways Analysis (IPA; Ingenuity Systems Inc., Redwood City, CA), Pathway Commons (Memorial Sloan-Kettering Cancer Center and the University of Toronto 2010), and Mouse and Online Mendelian Inheritance in Man [OMIM; National Center for Biotechnology Information (NCBI) 2010a]phenotype databases. In brief, a chemical-pathway perturbation score corresponds to the minimum AC_50_ for that chemical for any assay mapping to the pathway, where the chemical must show activity (AC_50_ or LEC) against at least five assay targets mapping to genes in the pathway. The calculation of these scores has been described in detail elsewhere (Judson et al. 2010). The perturbation scores are a method of aggregating assays into groups and providing a higher-level view of chemical activity across important signaling pathways.

For most of the ToxCast phase I chemicals, *in vivo* regulatory test guideline data are entered into ToxRefDB (Toxicity Reference Database; U.S. EPA 2010b). ToxRefDB contains standardized and computable data on 2-year cancer/chronic studies on rats and mice (Martin et al. 2009a), multigenerational reproductive studies on rats (Martin et al. 2009b), and prenatal developmental studies on rats and rabbits (Knudsen et al. 2009). The database includes standardized end points such as fetal body weight reduction, pubertal delays, skeletal malformations, and tumor formation in adult organs. The data are reported as LEL (lowest effect level) doses (milligrams per kilogram per day) for maternal, developmental, or categorical end points. All defect categories (skeletal, urogenital, neurosensory, etc.) include both malformations and variations, which, although they may be transitive delays in some cases (e.g., dilated renal pelvis), may still represent chemical effects. In total, there were 76 aggregated end points across the ToxRefDB prenatal studies. The ToxRefDB annotation system used internationally harmonized nomenclature (Knudsen et al. 2009; Makris et al. 2009; Wise et al. 1997) to create a thesaurus of 988 nonredundant terms that apply to maternal and developmental end points (Knudsen et al. 2009). The complete data set is available for download (U.S. EPA 2010b) and is also accessible via a web-searchable format.

**Figure 1:**
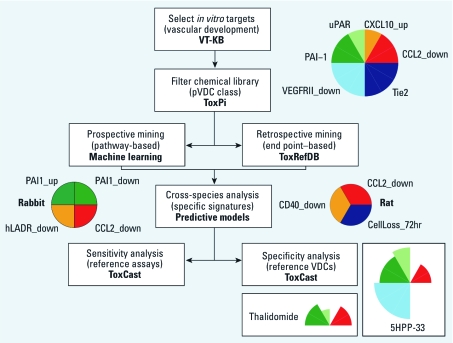
Work flow to identify pVDCs among 309 environmental chemicals. Assays critical to vascular development were identified by VT-KB, chemicals were ranked by their VBS against these vascular targets, and the ToxPi tool was used to filter/visualize the chemical library. Multivariate algorithms produced predictive signatures of species-specific vascular disruption, correlated with ToxRefDB *in vivo* end points in prenatal guideline studies. The chemical library was tested against additional reference *in vitro* assays, and reference antiangiogenic compounds (thalidomide and 5HPP-33) were tested against the pVDC signature.

*Work flow.* We probed the ToxCast data set for signatures of biological activity that could indicate the potential for disrupting blood vessel morphogenesis and remodeling (vasculogenesis/angiogenesis). The general work flow for identification, prioritization, hypothesis generation, and validation of pVDCs is shown in Figure 1. To select the relevant assays, we used the Virtual Tissues Knowledgebase (VT-KB; U.S. EPA 2010c) to extract and organize relevant facts from the scientific literature, using a dictionary of terms built with publicly available ontologies of genes, pathways, anatomy, clinical outcomes, and chemicals. We compiled a list of key words relevant to embryonic vascular formation [see Supplemental Material, Figure 1 (http://dx.doi.org/10.1289/ehp.1103412)] and cross-referenced these with > 300 ToxCast *in vitro* assay targets among 20 million abstracts in PubMed (NCBI 2010b). Relevancy of the assay targets was determined based on the number of cooccurrences with vascular key words and biological plausibility. Briefly, the vascular key words were first used to extract the subset of articles from PubMed that pertain to embryonic vascular development. This database of approximately 88,000 articles was named VasculoDB. All genes and proteins were then ranked by their number of occurrences within the abstracts from the VasculoDB and cross-referenced with the ToxCast assay targets. Each of the highest-ranking assay targets was used to perform further key word searches using all synonyms of that target [e.g., VEGFR2 (vascular endothelial growth factor receptor 2), KDR, VEGFRII, Flk1, etc.] and extract the relevant articles for manual full-text evaluation and curation. Biological plausibility was determined based on the scientific weight of evidence for each target being critical to and necessary for embryonic vascular development.

Chemicals were ranked for vascular-disruptive potential by their activity across the selected targets. This ranking was referred to as a vascular bioactivity score (VBS) and represented a weighted sum based on the log transform of the AC_50_/LEC values for the specified assay targets [for details, see Supplemental Material, pp. 4–5 (http://dx.doi.org/10.1289/ehp.1103412)]. The 123 chemicals that fell above the mean score for the entire phase I chemical landscape were designated as pVDCs. We tested reference thalidomide compounds with known antiangiogenic effects [thalidomide and 5-hydroxy-2-(2,6-diisopropylphenyl)-1H-isoindole-1,3-dione (5HPP-33); Sigma-Aldrich Corp., St. Louis, MO] in the identified subset of vascular assays (processed under the same conditions in relevant ToxCast assays) and calculated their VBS rankings. We identified additional assays with biochemical targets critical to vascular development and tested the ToxCast chemical library to determine if the rank order of activity corresponded with the predicted pVDC potential.

The subset of pVDCs with *in vivo* developmental toxicity data in ToxRefDB were analyzed for species-specific trends. We used a stepwise linear discriminant analysis (LDA) with 5-fold cross-validation to identify multivariate toxicity signatures based on significant associations between groups of pVDCs with developmental effects specific to rats or rabbits and the remainder of the ToxCast *in vitro* assays and pathway perturbation scores. Here the independent variables were the *in vitro* assay data, and the dependent variables, or predictions, were the groups of pVDCs with species-specific *in vivo* effects from animal testing data in ToxRefDB. The model with the best test-set balanced accuracy (BA; average of sensitivity and specificity) was chosen. For algorithm details, example queries, and equations, see Supplemental Material, pp. 5–7 (http://dx.doi.org/10.1289/ehp.1103412).

## Results

*Identifying pVDCs.* Six assay targets emerged as most relevant to embryonic blood vessel formation based on biological plausibility and prevalence in the VasculoDB literature. These targets have been previously identified, based on murine knockout models or significant associations between biomarker data and adverse pregnancy effects, as critical to proper embryonic blood vessel formation or maternal–fetal placental circulation. In order of descending influence (with corresponding references highlighting their importance to vascular development), these were down-regulation of the receptor tyrosine kinase (RTK) VEGFR2 (Drake et al. 2007); inhibition of the enzymatic activity of TIE2, an angiogenic RTK (Patan 1998); down-regulation of the proangiogenic chemokine CCL2 (Keeley et al. 2008); perturbation of the plasminogen-activating system (PAS) controlling extracellular matrix breakdown via up- or down-regulation of plasminogen activator inhibitor type 1 (PAI-1/SERPINE1) (Behzadian et al. 2003); up-regulation of the proinflammatory antiangiogenic chemokine CXCL10 (Romagnani et al. 2001); and perturbation of the PAS via up- or down-regulation of urokinase-type plasminogen activator receptor (uPAR/PLAUR) (D’Alessio and Blasi 2009). Other ToxCast assay targets broadly involved in cell signaling, such as transforming growth factor β (TGFβ), had occurrence levels comparable to these six features; however, the identified features were those deemed to be simultaneously highly relevant to embryonic vascular development and specific to those cell types that are directly involved in vasculo/angiogenic processes (endothelial cells, mural cells, inflammatory cells). In total, 23 unique assays were represented by the six target features, including one cell-free biochemical assay (Knudsen et al. 2011) and 22 assays combining eight primary human cell types with BioMAP (Biologically Multiplexed Activity Profiling) protein readouts from complex cell-culture mixtures to more closely simulate tissue biology (Houck et al. 2009).

**Figure 2:**
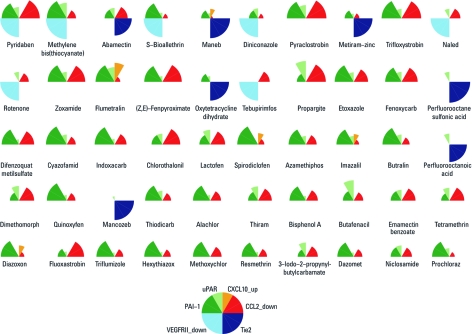
ToxPi visualization for top 50 pVDCs ranked by VBS across six *in vitro* targets: CCL2 down-regulation, CXCL10 up-regulation, uPAR up- and down-regulation, PAI‑1 up- and down-regulation, VEGFR2 down-regulation, and TIE2 binding.

A VBS was computed as a weighted sum of the activity across 23 assays representing the six prioritized vascular targets for each of 309 unique chemicals in the library. We found 123 of 309 chemicals with a VBS above the mean score for the entire phase I chemical landscape and provisionally labeled them as pVDCs. The top candidate was pyridaben (VBS = 6.9), and the mean cutoff corresponded to cyfluthrin (VBS = 1.48). The 186 chemicals that did not fall above the mean cutoff had a VBS range of 0–1.30. We ranked the 309 chemicals according to individual VBS and visualized the VBS ranking using ToxPi (Toxicological Priority Index) profiles (Reif et al. 2010). ToxPi results for the top 50 pVDCs are shown in Figure 2 [for profiles of the entire 309 ToxCast chemical library, see Supplemental Material, Figure 2 (http://dx.doi.org/10.1289/ehp.1103412)]. Each sector is normalized to show the relative effect of each chemical on each readout, and the overall profiles combine the relative weights across VBS targets into a single graphic for each pVDC.

*Reference thalidomide compounds.* Statistical models for pVDCs built from ToxCast data identified environmental chemicals with no previously known potential to disrupt vascular development. Thalidomide, a well-studied developmental toxicant, and its antiangiogenic analogue 5HPP-33 are known to disrupt blood vessel development (D’Amato et al. 1994; Noguchi et al. 2005; Sarkanen et al. 2011). These reference compounds were used to test the pVDC signature defined for the ToxCast chemical library. The corresponding VBS was derived in the same way from s AC_50_ and s LEC values (Table 1).

**Table 1:** Reference antiangiogenic compounds: activity across pVDC signature.

LEC (µM)											TIE2 AC_50_ (µM)
Chemical	VBS*a*	CCL2.	CXCL10-	uPAR1	PAI‑11	VEGFR2.					
5HPP-33		6.61		0.625		—		1.25		20		20		—
Thalidomide		1.76		0.625		—		2.5		40		—		—
Symbols: ., down-regulation; -, up-regulation; 1, up- and down-regulation; —, no change. **a**Summed activity across 23 assays, corresponding to the 6 assay targets shown here.														

As predicted, thalidomide and 5HPP-33 were active across most of the pVDC signature assay targets. Both compounds significantly down-regulated CCL2 with an LEC of 0.625 µM in a coculture of peripheral blood mononuclear cells and endothelial cells. Thalidomide up-regulated PAI-1 in fibroblasts at 40 µM, whereas 5HPP-33 down-regulated PAI-1 in both fibroblasts and bronchial epithelial cells (LEC = 20 µM). 5HPP-33 up-regulated uPAR in bronchial epithelial cells at 1.25 µM, and thalidomide up-regulated uPAR in endothelial cells at 2.5 µM. 5HPP-33 also had effects on uPAR in the endothelial cell system at 10 µM. Neither compound affected the CXCL10 signal assay or produced an AC_50_ in the TIE2 assay. Both compounds, however, fell above the mean VBS cutoff and would be classified as pVDCs based on *in vitro* activity for vascular targets identified by the ToxCast data and chemicals. Thalidomide had a lower VBS and would be predicted as a moderate vascular disruptor, whereas the analog designed specifically to be antiangiogenic, 5HPP-33, scored the second highest VBS among all 311 compounds tested (ToxPi visualization shown in Figure 2). Thus, our findings with reference compounds known to be antiangiogenic support the contention that VBS is an effective metric for vascular disruption.

*Reference vascular targets.* We expanded the initial ToxCast assay portfolio to include additional biochemical assay targets known to be critical in vascular development: VEGF receptors VEGFR1, VEGFR2, and VEGFR3; the arterial vessel marker EphB2 (ephrin-B2); and PI3Ka (phosphatidylinositol 3-kinase) and PTEN (phosphatase and tensin homolog), both of which have been shown to govern normal vascular development (Chappell et al. 2009; Drake et al. 2007; Eichmann et al. 2008; Hamada et al. 2005). We hypothesized that the ToxCast chemicals predicted to be pVDCs would have greater activity on these targets than that shown by the remainder of the chemical library. The chemical library was run against these assays, as was a repeat of the TIE2 assay from the original data series (Table 2). Of the 309 compounds, 14 had one or more AC_50_ hits across these targets. Of these candidates, 11 were previously classified as pVDCs based on the original portfolio, which involved one biochemical assay (TIE2) and five assays from cell–cell signaling platforms. The chemicals in Table 2 are ranked based on their VBS. The 11 pVDCs resulted in 42 AC_50_ values across these critical vascular targets, with an average AC_50_ of 16 µM, whereas the three non-pVDCs resulted in four AC_50_ values, with an average value of 28 µM. The results from these assays revealed a trend whereby pVDCs with a higher VBS had lower AC_50_ values (30 values ≤ 20 µM) across a wider variety of vascular targets, whereas compounds that did not make the pVDC cutoff had higher AC_50_ values (one value ≤ 20 µM) for a more limited number of targets. These additional data for more targets in vascular pathways further demonstrated that VBS is an effective tool identifying vascular disruptors.

**Table 2:** Chemical activities (AC_50_) across biochemical targets critical to vascular development.

AC_50_ (µM)							
ToxCast chemical name	VBS	VEGFR1	VEGFR2	VEGFR3	TIE2	EphB2	PI3Ka	PTEN
Predicted pVDCs from ToxCast data									
Abamectin		5.19				38.8	17	6.4	
Maneb		4.69	4.1	31	12	8.08	23		0.9
Metiram-zinc		4.35	6.6	41	22	15		29	12
Oxytetracycline dihydrate		3.85		19	6.5	15		6.2	1.6
Perfluorooctanesulfonic acid		3.48	8.2	50	8	4.43		6.5	17
Perfluorooctanoic acid		3.04				31.9			
Mancozeb		3.00	19	5.9	1.3	10.4	21	20	0.23
Emamectin benzoate		2.91					21	7.9	2.1
2,2-bis-(*p*-Hydroxyphenyl)-1,1,1-trichloroethane		2.28						28	
Diclosulam		2.09							50
Milbemectin		1.49					20	11	12
Predicted non-pVDCs from ToxCast data									
Captan		1.14							35
Cyclanilide		0.21					50		3.9
Sethoxydim		0.00							22

*Correlations with* in vivo *animal data.* Most ToxCast chemicals have corresponding *in vivo* bioassay data in ToxRefDB (Knudsen et al. 2009; Martin et al. 2009a, 2009b), a standardized and computable database that includes 76 aggregated end points across rat and rabbit prenatal developmental toxicity studies (U.S. EPA 2010b). The candidate pVDCs were cross-referenced to ToxRefDB developmental effects [for a list of pVDCs (*n* = 17) without prenatal guideline study data in either species, see Supplemental Material, Table 1 (http://dx.doi.org/10.1289/ehp.1103412)]. Many of those chemicals, such as imazalil and perfluorooctanoic acid, are known from the literature to have mammalian developmental toxicity (Lau et al. 2004; Zega et al. 2009), and these data were captured by the VT-KB and reported, where applicable [for a list of pVDCs that did not have a recorded developmental effect or result in pregnancy-related fetal loss in rat or rabbit (*n* = 7), see Supplemental Material, Table 2]. In addition to the correlations with end points from the prenatal literature, toxicity was exhibited across a wide range of biological systems in chronic and multigenerational guideline studies; the most common end points recorded were liver and kidney effects.

For pVDCs with data in both rat and rabbit (*n* = 84), adverse effects directly on the embryo were seen with 68% of chemicals. In rabbits, the most numerous end points (*k*) were embryonic fetal loss (*k* = 23), skeletal axial defects (*k* = 17), and fetal weight reduction (*k* = 15). In rats, the most prevalent end points were skeletal axial defects (*k* = 40), fetal weight reduction (*k* = 30), embryonic fetal loss (*k* = 21), and skeletal appendicular defects (*k* = 16). The category of skeletal defects potentially shows high representation because bone elements, such as vertebrae, femur, and ribs, are entered into the database as individual targets and further annotated by descriptions such as absent, incomplete ossification, or misshapen. In the ToxRefDB ontology, maternal effects are recorded separately. In cases of increased resorptions and fetal losses, it is not possible to know whether these effects were maternal or embryo mediated. Pregnancy-related fetal loss (all expressions of fetal wastage, including preimplantation loss, implantation failure, resorptions, and fetal death) was a distinct category entered into ToxRefDB that may also be indicative of embryotoxicity, and if this additional end point is included, the number of potentially developmentally toxic pVDCs with prenatal data in both rats and rabbits increased from 68% to 92%. This was the most prevalent end point in the rabbit (*k* = 32) and also occurred in the rat (*k* = 20).

*Species-specific signatures.* We observed a striking difference between the subsets of pVDCs active in pregnant rats versus rabbits, which raised two important questions about predictive modeling useful for informing human health risk assessments. First, can the same suite of *in vitro* assays based primarily on human cells and biochemical targets provide a signature that distinguishes chemicals developmentally toxic in rabbits from those developmentally toxic in rats? Second, what distinguishes pathway-level perturbations by chemicals that affect one species differently from the other?

Among pVDCs tested in both species and entered into ToxRefDB, 22 candidates exhibited developmental toxicity in the rabbit only (Table 3), and 21 chemicals resulted in developmental phenotypes in the rat only (Table 4). Each subset of species-specific pVDCs was run through a stepwise LDA to determine commonalities among their bioactivity profiles and to identify susceptible systems that can account for the cross-species differences. In each case we considered the set of pVDCs that were tested in both species, where the model positives were those that showed effects specific to one species and the negatives were the remainder of the set. The algorithm was run several times with varying feature sets allowing for linear combinations of *in vitro* assays, *in vivo* data, and pathway perturbation scores, and we chose the model with best cross-validation test BA. Running the algorithm with the same feature set several times did not produce different BAs, so the model was assumed to be stable.

**Table 3:** pVDCs with rabbit-specific effects in ToxRefDB prenatal studies.

Chemical	VBS	Developmental phenotype
Methylene bis(thiocyanate)		6.55		Maternal pregnancy loss
Trifloxystrobin		4.26		Skeletal: axial
Propargite		3.79		Skeletal: axial
Etoxazole		3.66		Skeletal: axial
Fenoxycarb		3.60		Skeletal: axial
Azamethiphos		3.10		Maternal pregnancy loss
Quinoxyfen		3.02		Maternal pregnancy loss
Butafenacil		2.94		Embryo fetal loss
Dazomet		2.61		General fetal pathology; embryo fetal loss; skeletal: axial
Rimsulfuron		2.52		Embryo fetal loss; maternal pregnancy loss
Dichlorvos		2.34		Maternal pregnancy loss
Bensulide		2.17		Maternal pregnancy loss
Flumiclorac-pentyl		2.11		Maternal pregnancy loss
Diclosulam		2.09		Maternal pregnancy loss
Propetamphos		2.08		Embryo fetal loss
Butachlor		1.99		Fetal weight reduction; embryo fetal loss; maternal pregnancy loss
Dicofol		1.92		Maternal pregnancy loss
Oxyfluorfen		1.82		Embryo fetal loss; maternal pregnancy loss
Famoxadone		1.82		Embryo fetal loss; maternal pregnancy loss
Flufenpyr-ethyl		1.73		Maternal pregnancy loss
Dicrotophos		1.59		Fetal weight reduction; maternal pregnancy loss
Carboxin		1.56		Maternal pregnancy loss

**Table 4:** pVDCs with rat-specific effects in ToxRefDB prenatal studies.

Chemical	VBS	Developmental phenotype
Diniconazole		4.57		General fetal pathology; embryo fetal loss; maternal pregnancy loss; skeletal: appendicular, axial; urogenital: renal
Naled		4.22		Embryo fetal loss; trunk: body wall
(*Z*,*E*)-Fenpyroximate		3.88		Skeletal: axial
Cyazofamid		3.34		Skeletal: axial
Chlorothalonil		3.28		Embryo fetal loss; maternal pregnancy loss
Lactofen		3.18		Fetal weight reduction; maternal pregnancy loss; skeletal: appendicular; skeletal: axial
Spirodiclofen		3.15		Urogenital: renal
Thiodicarb		2.97		Fetal weight reduction, general fetal pathology; embryo fetal loss; maternal pregnancy loss; skeletal: axial
Alachlor		2.97		Fetal weight reduction; embryo fetal loss
Emamectin benzoate		2.91		Fetal weight reduction; skeletal: appendicular, axial, cranial
Fluoxastrobin		2.79		Skeletal: appendicular
Hexythiazox		2.78		Skeletal: appendicular
Tetraconazole		2.23		General fetal pathology; skeletal: axial; urogenital: renal, ureteric
Prodiamine		2.19		Neurosensory: eye
Fenpropathrin		2.18		Maternal pregnancy loss
Acetochlor		2.16		Fetal weight reduction; general fetal pathology; embryo fetal loss; maternal pregnancy loss; skeletal: axial
Prallethrin		2.11		Maternal pregnancy loss
Thiazopyr		2.00		Skeletal: axial
Fludioxonil		1.82		Urogenital: renal, ureteric
Profenofos		1.71		Maternal pregnancy loss
Metolachlor		1.57		Fetal weight reduction; embryo fetal loss; maternal pregnancy loss

Remarkably, the statistical model predicted that the PAS was a significant target for pVDCs active in the rabbit but not rat studies. The best predictive signature was capable of identifying with 80% accuracy rabbit-specific developmental toxicants that may be working via a vascular-disruptive mechanism (test BA = 0.8 over 20 iterations). Table 5 shows the model features, which include down-regulation of PAI-1 in a complex culture assay of bronchial epithelial cells and a group of pathways from databases such as GO, IPA, and KEGG. Many of the pathways being targeted by these compounds are related to interactions with the extracellular matrix and the PAS and include processes such as regulation of angiogenesis, fibrinolysis, collagen binding, blood coagulation, and transmembrane receptor activity. Other perturbed pathways, such as regulation of apoptosis and p53 signaling, pertain to cellular survival and programmed cell death critical to proper embryonic development. When run against a feature set consisting of only the ToxCast assays, the signature had lower predictivity (test BA = 0.6) but was still focused on elements of the PAS, with down-regulation and up-regulation of PAI-1 emerging as the two strongest components. This signature is represented by the rabbit ToxPi in Figure 1. The pattern of *in vivo* end points caused by the rabbit-specific pVDCs (Table 3) was nearly uniform, with 18 of 22 (82%) resulting in either embryonic loss or maternal pregnancy loss and 5 of 22 resulting in skeletal axial defects (dazomet affected both end points and caused general fetal pathology). Fetal weight reduction was also due to butachlor and dicrotophos in ToxRefDB (U.S. EPA 2010b).

**Table 5:** Multivariate toxicity signature: pVDCs with rabbit-specific effects in ToxRefDB prenatal studies.

Descriptor	Result
Model statistics		
Learner		LDA
CV		5-fold
BA train		0.86
BA test		0.8
Best sensitivity		0.84
Best specificity		0.84
Best AUC		0.86
Model features		
Pathways		
Gene		SERPINE1 (PAI‑1); MMP9
GO: Process		Regulation of angiogenesis; fibrinolysis; blood coagulation; positive regulation of apoptosis
KEGG		p53 signaling pathway; complement coagulation cascades
IPA		Coagulation system; acute phase response signaling
GO: Function		Serine type endopeptidase activity; transmembrane receptor activity; collagen binding
GO: Component		Extracellular region; integral to membrane
ToxCast assays		BSK:BE3C (bronchial epithelial cells): PAI‑1 down
Abbreviations: AUC, area under receiver operating characteristic curve; BA, balanced accuracy; CV, cross validation; MMP9, matrix metalloproteinase 9. Pathways were output from different annotations, including GO (2010), KEGG (Kanehisa Laboratories 2010), and PathwayCommons (Memorial Sloan-Kettering Cancer Center and the University of Toronto 2010).		

In contrast, pVDCs that were active in the rat but not in rabbit prenatal studies (Table 4) were significantly associated with inflammatory response targets. The signature was able to identify with 90% accuracy rat-specific developmental toxicants that may be acting via a vascular-disruptive mechanism (cross-validation BA = 0.9). The model features (Table 6) consisted of three assays: CCL2 down-regulation in a complex coculture of peripheral blood mononuclear cells and endothelial cells, CD40 down-regulation in the same system, and a cell viability assay in the HepG2 human hepatocellular carcinoma cell line. This model was produced by a feature set containing only the ToxCast assays. When run against a feature set including pathway perturbation scores, the same signature is produced with one additional feature, the GO pathway “Component: integral to membrane,” and a slightly lower BA = 0.84 (data not shown). These compounds appear to be preferentially targeting inflammatory signaling, specifically via the angiogenic chemokine CCL2 and CD40, a tumor necrosis factor receptor family protein that controls a variety of immune-related processes and is expressed by vascular endothelial cells and macrophages, among others. The downstream effects of perturbing CD40/chemokine signaling involve interruption of cell–cell adhesion and proliferation, which could correlate with the cell loss observed in HepG2 cells at the 72-hr time point. *In vivo* end points caused by the rat-specific pVDCs were more diverse, with fetal loss being less common (10 of 21) than in the rabbit and a wider variety of phenotypes, with the most common being axial and appendicular skeletal defects. We also observed urogenital end points (due to diniconazole, spirodiclofen, tetraconazole, and fludioxonil) and fetal weight reduction in ToxRefDB (U.S. EPA 2010b).

**Table 6:** Multivariate toxicity signature: pVDCs with rat-specific effects in ToxRefDB prenatal studies.

Descriptor	Result
Model statistics	
Learner	LDA
CV	5-fold
BA train	0.9
BA test	0.9
Best sensitivity	0.9
Best specificity	0.9
Best AUC	0.9
Model features	
ToxCast assays	BSK:SAg (peripheral blood mononuclear cells + endothelial cells): CCL2 down, CD40 down
	CLM (HepG2 human hepatocellular carcinoma cell line): cell loss 72-hr time point
Abbreviations: AUC, area under receiver operating characteristic curve; BA, balanced accuracy; CV, cross-validation.	

## Discussion

These results provide a novel ranking of environmental chemicals based on potential to disrupt critical targets in vascular development and suggest a strong association with the potential for adverse developmental effects in pregnant rats or rabbits. This finding frames the hypothesis that vascularization of the embryo–placenta is a common biological target for the developmental toxicity of some environmental chemicals that act as pVDCs. The two most prevalent effects in the animal prenatal testing, skeletal malformation and fetal loss, further suggest that pVDCs act on different aspects of vascular signaling, including the VEGF pathway, the PAS, and the chemokine signaling network. Deeper examination of ToxCast chemicals also revealed a species-specific *in vivo* response in the inflammatory signaling network (rats) and the PAS (rabbits). It is noteworthy that the reference compounds tested here (thalidomide, 5HPP-33) produced results consistent with the observed vascular disruption signature. This study demonstrates the use of rapid *in vitro* testing to rank and prioritize environmental chemical libraries to identify compounds with the potential for vascular disruption. We developed a phenomenological vascular-disruptive toxicity signature based on short-term assay information and showed that the most compounds identified by this signature also caused *in vivo* developmental toxicity.

Prenatal guideline studies *in vivo* record predominantly apical end points with insufficient detail to determine whether the observed developmental toxicity is via vascular disruption or some other mechanism. One would expect that most pVDCs would be potential developmental toxicants, as is the case here (92%), but not that all developmental toxicants would be pVDCs. Of the remaining ToxCast phase I compounds (non-pVDCs) with prenatal guideline studies in both species (*n* = 130), 75% have direct embryonic effects or pregnancy-related fetal loss recorded in ToxRefDB, and we would predict that those effects are via a mechanism other than vascular disruption. We were unable to find any statistically significant concordance between the potency of the AC_50_ values *in vitro* and the incidence or pattern of developmental effects due to the ToxCast compounds, pVDC or otherwise (Sipes et al. 2011).

Many vascular-active developmental toxicants from the literature show concordance with the signaling pathways and specific targets identified in this analysis. The first antiangiogenic compound to be assessed in clinical trials, TNP-470 (*O*-[chloroacetyl-carbamoyl]fumagillol), resulted in spontaneous resorption and intrauterine growth restriction when administered early or late in murine pregnancy, respectively (Rutland et al. 2005). Targeted examination of TNP-470’s effect on the ocular vasculature revealed a significant decrease in VEGF expression and VEGFR2 phosphorylation (Joussen et al. 2001; Satchi-Fainaro et al. 2005). Placental vascular remodeling was suppressed in rats after exposure to the developmental toxicant 2,3,7,8-tetrachlorodibenzo-*p*-dioxin (TCDD) and was significantly correlated with decreased expression of *TIE2* mRNA (Ishimura et al. 2009). Angiostatin is a naturally occurring product of the PAS from the cleavage of plasminogen with strong inhibitory effects on blood vessel formation *in vivo* (Jing et al. 2004). Thalidomide is an antiangiogenic compound that has been studied for > 40 years and was shown here to target inflammatory signaling and extracellular matrix breakdown via the PAS. This corresponds to the pattern of genetic perturbations of vascular signaling found in a recent study in pregnant monkeys (Ema et al. 2010). The thalidomide analog 5HPP-33 was shown to have potent antiangiogenic activity in a human umbilical vein endothelial cell assay (~ 60% inhibition of tube formation), whereas the parent compound had moderate activity (~ 30% inhibition of tube formation) (Noguchi et al. 2005). When tested against our signature, both compounds were classified as pVDCs; however, 5HPP-33 was predicted to be more strongly antiangiogenic than the parent compound, as expected.

**Figure 3:**
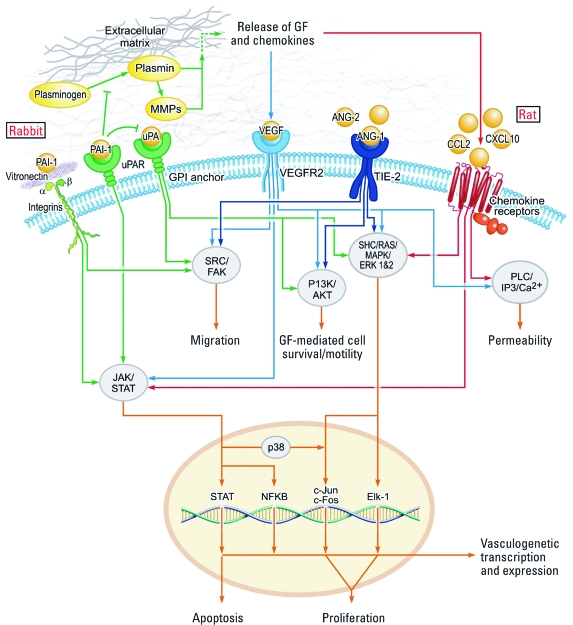
Vascular developmental signaling pathways identified by pVDC signature and hypothesized species-specific target sites. Rabbit-specific developmental toxicants potentially target the PAS (e.g., PAI‑1, uPAR), and rat-specific developmental toxicants potentially target inflammatory chemokine signaling (e.g., CXCL10, CCL2). The VEGF and ANG/TIE2 pathways are critical pathways associated with developmental toxicity across species. Abbreviations: ANG, angiopoietin; Elk‑1, ETS-like transcription factor 1; GPI, glycosylphosphatidylinositol; JAK/STAT, Janus kinase/signal transducer and activator of transcription; MMP, matrix metalloproteinase; NFKB, nuclear factor kappa B; PLC/IP3, phospholipase C/inositol triphosphate; P13K/AKT, phosphatidylinositol 3-kinase/protein kinase B; SHC/RAS/MAPK/ERK 1&2, Src homology 2 domain containing proteins/RAt sarcoma family/mitogen-activated protein kinases/extracellular-signal-regulated kinases 1 and 2; SRC/FAK, sarcoma tyrosine kinase/focal adhesion kinase.

An intriguing result of this analysis is the emergence of a possible species-specific signature that may shed light on the mechanisms underlying differential effects in animal studies. A subset of chemicals with rat-specific developmental toxicity correlated with down-regulation of proinflammatory chemokine assays, whereas the subset of chemicals with rabbit-specific activity resulted in up-regulation of these signals. We observed a bias toward targets in the PAS and extracellular regions by rabbit-specific chemicals, with greater bioactivity across assay end points such as PAI-1. The observed developmental toxicity also showed a differential response across species, with higher incidences of skeletal deformation in rat studies and of prenatal death in rabbit studies. Recent work has shown that a functional overlap of plasminogen and matrix metalloproteinases (MMPs) regulates placental vascularization (Solberg et al. 2003). Additionally, the hemostatic challenge of placentation requires a shift from an anti- to a procoagulant state (Li and Huang 2009), and the rabbit-specific pVDCs were correlated with several blood coagulation pathways. We speculate that pVDCs have a strong effect on placentation in the rabbit, leading to prenatal death, whereas the vascular-disruptive mechanism in the rat may be acting farther downstream via the inflammatory system and targeting vessel remodeling of the forming skeletal system. The signature derived here would have predicted that thalidomide, because of its impact on the PAS, would affect the developing rabbit. Such has been confirmed many times *in vivo*, although the mechanism was attributed to redox imbalance rather than antiangiogenesis (Hansen et al. 2001). To our knowledge, 5HPP-33 has not been tested in pregnant rats or rabbits, but the VEGFR2 signature effect would lead us to predict that it would interrupt vascular development across species. Figure 3 depicts an overview of vascular developmental signaling involved in the pVDC signature and the proposed species-specific target pathways, where chemicals preferentially affecting the rabbit may be acting on the PAS and those preferentially affecting the rat may be more strongly perturbing inflammatory signaling.

Although there is no doubt that conservation of vascular developmental pathways across species exists, the relative importance of these pathways may differ in each species. Similarly, the consequences of disrupting these pathways may be species specific. Species-specific differences in pharmacokinetics could contribute to variations in developmental effects due to chemical insult, as well as affecting *in vitro* to *in vivo* predictions. Because of intercorrelation among assays and pathways, there is a potential for overfitting of the models. Continued investigation is required to increase our understanding of this potential connection between developmental toxicity and vascular disruption and further delineate which elements of these signatures are most useful in predicting and modeling potential effects in humans.

The pVDC toxicity signature represents an important tool in evaluating the vascular-disruptive potential of environmental chemicals. Although ToxCast HTS data and other information may not yet be sufficient to predict developmental toxicity in animals and humans, it is providing unique and valuable mechanistic information on prototypical toxicity pathways such as vascular disruption that could assist in chemical prioritization and further targeted testing.

## Supplemental Material

(396 KB) PDF

## References

[r1] Behzadian MA, Windsor LJ, Ghaly N, Liou G, Tsai NT, Caldwell RB (2003). VEGF-induced paracellular permeability in cultured endothelial cells involves urokinase and its receptor.. FASEB J.

[r2] Chappell JC, Taylor SM, Ferrara N, Bautch VL (2009). Local guidance of emerging vessel sprouts requires soluble Flt-1.. Dev Cell.

[r3] Collins FS, Gray GM, Bucher JR (2008). Toxicology. Transforming environmental health protection.. Science.

[r4] Czirok A, Zamir EA, Szabo A, Little CD (2008). Multicellular sprouting during vasculogenesis.. Curr Top Dev Biol.

[r5] D’Alessio S, Blasi F. (2009). The urokinase receptor as an entertainer of signal transduction.. Front Biosci.

[r6] D’Amato RJ, Loughnan MS, Flynn E, Folkman J (1994). Thalidomide is an inhibitor of angiogenesis.. Proc Natl Acad Sci USA.

[r7] Dix DJ, Houck KA, Martin MT, Richard AM, Setzer RW, Kavlock RJ (2007). The ToxCast program for prioritizing toxicity testing of environmental chemicals.. Toxicol Sci.

[r8] Drake CJ, Fleming PA, Argraves WS (2007). The genetics of vasculogenesis.. Novartis Found Symp.

[r9] Eichmann A, Bouvrée K, Pardanaud L (2008). Vasculogenesis and angiogenesis in development.

[r10] Ema M, Ise R, Kato H, Oneda S, Hirose A, Hirata-Koizumi M (2010). Fetal malformations and early embryonic gene expression response in cynomolgus monkeys maternally exposed to thalidomide.. Reprod Toxicol.

[r11] Ferrara N. (2004). Vascular endothelial growth factor: basic science and clinical progress.. Endocr Rev.

[r12] GO (2010). The Gene Ontology.. http://www.geneontology.org/.

[r13] Hamada K, Sasaki T, Koni PA, Natsui M, Kishimoto H, Sasaki J (2005). The PTEN/PI3K pathway governs normal vascular development and tumor angiogenesis.. Genes Dev.

[r14] Hansen JM, Choe HS, Carney EW, Harris C (2001). Differential antioxidant enzyme activities and glutathione content between rat and rabbit conceptuses.. Free Radic Biol Med.

[r15] Hanson DR Gottesman II 2005 Theories of schizophrenia: a genetic-inflammatory-vascular synthesis. BMC Med Genet 6 7; doi:10.1186/1471-2350-6-7 [Online 11 February 2005] 15707482 PMC554096

[r16] Houck KA, Dix DJ, Judson RS, Kavlock RJ, Yang J, Berg EL (2009). Profiling bioactivity of the ToxCast chemical library using BioMAP primary human cell systems.. J Biomol Screen.

[r17] Hoyme HE, Jones KL, Dixon SD, Jewett T, Hanson JW, Robinson LK (1990). Prenatal cocaine exposure and fetal vascular disruption.. Pediatrics.

[r18] Ishimura R, Kawakami T, Ohsako S, Tohyama C. (2009). Dioxin-induced toxicity on vascular remodeling of the placenta.. Biochem Pharmacol.

[r19] Ito T, Ando H, Suzuki T, Ogura T, Hotta K, Imamura Y (2010). Identification of a primary target of thalidomide teratogenicity.. Science.

[r20] Jing S, Sarah XZ, Chunkui S, James F, Jian-xing M (2004). The effect of angiostatin on vascular leakage and VEGF expression in rat retina.. FEBS Lett.

[r21] Joussen AM, Beecken WD, Moromizato Y, Schwartz A, Kirchhof B, Poulaki V (2001). Inhibition of inflammatory corneal angiogenesis by TNP-470.. Invest Ophthalmol Vis Sci.

[r22] Judson RS, Houck KA, Kavlock RJ, Knudsen TB, Martin MT, Mortensen HM (2010). *In vitro* screening of environmental chemicals for targeted testing prioritization: the ToxCast project.. Environ Health Perspect.

[r23] Kanehisa Laboratories (2010). KEGG: Kyoto Encyclopedia of Genes and Genomes.. http://www.genome.jp/kegg/.

[r24] Keeley EC, Mehrad B, Strieter RM (2008). Chemokines as mediators of neovascularization.. Arterioscler Thromb Vasc Biol.

[r25] Kitambi SS, McCulloch KJ, Peterson RT, Malicki JJ (2009). Small molecule screen for compounds that affect vascular development in the zebrafish retina.. Mech Dev.

[r26] Klauber N, Parangi S, Flynn E, Hamel E, D’Amato RJ (1997). Inhibition of angiogenesis and breast cancer in mice by the microtubule inhibitors 2-methoxyestradiol and taxol.. Cancer Res.

[r27] Knudsen T, Houck K, Sipes N, Singh A, Judson R, Martin M (2011). Activity profile of 320 ToxCast™ chemicals evaluated across 292 biochemical targets.. Toxicology.

[r28] Knudsen TB, Martin MT, Kavlock RJ, Judson RS, Dix DJ, Singh AV (2009). Profiling the activity of environmental chemicals in prenatal developmental toxicity studies using the U.S. EPA’s ToxRefDB.. Reprod Toxicol.

[r29] Lau C, Butenhoff JL, Rogers JM (2004). The developmental toxicity of perfluoroalkyl acids and their derivatives.. Toxicol Appl Pharmacol.

[r30] Li M, Huang SJ (2009). Innate immunity, coagulation and placenta- related adverse pregnancy outcomes.. Thromb Res.

[r31] Makris SL, Solomon HM, Clark R, Shiota K, Barbellion S, Buschmann J (2009). Terminology of developmental abnormalities in common laboratory mammals (version 2).. Reprod Toxicol.

[r32] Martin MT, Judson RS, Reif DM, Kavlock RJ, Dix DJ (2009a). Profiling chemicals based on chronic toxicity results from the U.S. EPA ToxRef Database.. Environ Health Perspect.

[r33] Martin MT, Mendez E, Corum DG, Judson RS, Kavlock RJ, Rotroff DM (2009b). Profiling the reproductive toxicity of chemicals from multigeneration studies in the toxicity reference database.. Toxicol Sci.

[r34] Mellin GW, Katzenstein M (1962). The saga of thalidomide. Neuropathy to embryopathy, with case reports of congenital anomalies.. N Engl J Med.

[r35] Memorial Sloan-Kettering Cancer Center and the University of Toronto (2010). Pathway Commons.. http://www.pathwaycommons.org/pc/.

[r36] National Research Council (2007). Toxicity Testing in the 21st Century: A Vision and a Strategy.

[r37] NCBI (National Center for Biotechnology Information) (2010a). OMIM: Online Mendelian Inheritance in Man.. http://www.ncbi.nlm.nih.gov/omim.

[r38] NCBI (National Center for Biotechnology Information) (2010b). PubMed.. http://www.ncbi.nlm.nih.gov/pubmed/.

[r39] Noguchi T, Fujimoto H, Sano H, Miyajima A, Miyachi H, Hashimoto Y. (2005). Angiogenesis inhibitors derived from thalidomide.. Bioorg Med Chem Lett.

[r40] Patan S. (1998). TIE1 and TIE2 receptor tyrosine kinases inversely regulate embryonic angiogenesis by the mechanism of intussusceptive microvascular growth.. Microvasc Res.

[r41] Reif DM, Martin MT, Tan SW, Houck KA, Judson RS, Richard AM (2010). Endocrine profiling and prioritization of environmental chemicals using ToxCast data.. Environ Health Perspect.

[r42] Romagnani P, Annunziato F, Lasagni L, Lazzeri E, Beltrame C, Francalanci M (2001). Cell cycle-dependent expression of CXC chemokine receptor 3 by endothelial cells mediates angiostatic activity.. J Clin Invest.

[r43] Rutland CS, Mukhopadhyay M, Underwood S, Clyde N, Mayhew TM, Mitchell CA (2005). Induction of intrauterine growth restriction by reducing placental vascular growth with the angioinhibin TNP-470.. Biol Reprod.

[r44] Sarkanen J-R Mannerströäm M Vuorenpää H Uotila J Ylikomi T Heinonen T 2011 Intra-laboratory pre-validation of a human cell based *in vitro* angiogenesis assay for testing angiogenesis modulators. Front Pharmacol 1 147; doi:10.3389/fphar.2010.00147 [Online 20 January 2011] 21779245 PMC3134867

[r45] Satchi-Fainaro R, Mamluk R, Wang L, Short SM, Nagy JA, Feng D (2005). Inhibition of vessel permeability by TNP-470 and its polymer conjugate, caplostatin.. Cancer Cell.

[r46] Sipes NS Martin MT Reif DM Kleinstreuer NC Judson RS Singh AV 2011 Predictive models of prenatal developmental toxicity from ToxCast high-throughput screening data. Toxicol Sci; doi:10.1093/toxsci/kfr220 [Online 26 August 2011] 21873373

[r47] Solberg H, Rinkenberger J, Dano K, Werb Z, Lund LR (2003). A functional overlap of plasminogen and MMPs regulates vascularization during placental development.. Development.

[r48] Therapontos C, Erskine L, Gardner ER, Figg WD, Vargesson N (2009). Thalidomide induces limb defects by preventing angiogenic outgrowth during early limb formation.. Proc Natl Acad Sci USA.

[r49] Tideman E, Marsal K, Ley D. (2007). Cognitive function in young adults following intrauterine growth restriction with abnormal fetal aortic blood flow.. Ultrasound Obstet Gynecol.

[r50] U.S. EPA (U.S. Environmental Protection Agency) (2010a). ToxCast™.. http://www.epa.gov/ncct/toxcast.

[r51] U.S. EPA (U.S. Environmental Protection Agency) (2010b). ToxRefDB.. http://www.epa.gov/ncct/toxrefdb/.

[r52] U.S. EPA (U.S. Environmental Protection Agency) (2010c). Virtual Tissues Knowledgebase (VT-KB).. http://www.epa.gov/ncct/v-Embryo/discovery.html.

[r53] Vargesson N. (2009). Thalidomide-induced limb defects: resolving a 50-year-old puzzle.. Bioessays.

[r54] Wise LD, Beck SL, Beltrame D, Beyer BK, Chahoud I, Clark RL (1997). Terminology of developmental abnormalities in common laboratory mammals (version 1).. Teratology.

[r55] Zega G, De Bernardi F, Groppelli S, Pennati R. (2009). Effects of the azole fungicide Imazalil on the development of the ascidian *Ciona intestinalis* (Chordata, Tunicata): morphological and molecular characterization of the induced phenotype.. Aquat Toxicol.

