# Supplementary material for: Environmental Impact on Vascular Development Predicted by High-Throughput Screening
Source: Environ Health Perspect. 2011 Jul 25;119(11):1596–603. doi: 10.1289/ehp.1103412 (PMC3226499; doi:10.1289/ehp.1103412)
Supplement: (396 KB) PDF [file ehp.1103412.s001.pdf]

Supplemental Material:

Environmental Impact on Vascular Development Predicted by High Throughput Screening

Nicole C. Kleinstreuer[1], Richard S. Judson[1], David M. Reif[1], Nisha S. Sipes[1], Amar V. Singh[2], Kelly J. Chandler[1,3], Rob DeWoskin[4], David J. Dix[1], Robert J. Kavlock[1] and Thomas B. Knudsen[1]

[1]National Center for Computational Toxiciology, Office of Research and Development, U.S. Environmental Protection Agency, Research Triangle Park, NC, USA, [2]Lockheed Martin, RTP, NC, USA, [3]National Health and Environmental Effects Research Lab and [4]National Center for Environmental Assessment, Office of Research and Development, U.S. Environmental Protection Agency, RTP, NC, USA

Corresponding Author:

Nicole C. Kleinstreuer, PhD

National Center for Computational Toxicology (B205-01)

Office of Research & Development, U.S. Environmental Protection Agency

Research Triangle Park, NC 27711

Email: kleinstreuer.nicole@epa.gov

Phone 919 541 5776 Fax 919 541 1194

Disclaimer: *The views expressed in this article are those of the authors and do not necessarily reflect the views or policies of the U.S. Environmental Protection Agency. Mention of trade names or commercial products does not constitute endorsement or recommendation for use.*

Table of Contents:

Virtual Tissues Knowledge Base	4
Vascular Bioactivity Score	4-5
Multivariate Modeling	5-7
SM.Figure 1	8
SM.Figure 2	9
SM.Table 1	10-12
SM.Table 2	13-14

Virtual Tissues Knowledgebase (VT-KB)

VT-KB represents a flexible platform to extract and organize relevant facts from the existing body of scientific literature. Briefly, a vocabulary of terms was built to describe concepts relevant to health and disease using publicly available ontologies including genes, pathways, anatomy, clinical outcomes, and chemicals. We compiled a list of keywords relevant to embryonic vascular formation (Supplemental Figure 1), and varying combinations of these and like terms were cross-referenced in the VT-KB with the list of ToxCast *in vitro* assay targets. Supplemental Figure 1 shows the example of cross-referencing the vascular developmental keywords with the VEGFR2 receptor and the many synonyms (KDR, Flk-1, etc) found in the literature. Initial results showed a high incidence of publications related to tumor neo-vascularization; therefore, the query was filtered using NOT logic with the keyword "cancer". The keyword search results (over 20 million PubMed abstracts searched) were stored in a MySQL database for statistical analyses to summarize relationships and map them to biological concepts and pathways.

Vascular Bioactivity Score (VBS)

A weighted score (VBS) was created for each chemical across the six *in vitro* targets with the highest ranking, based on the log transform of the AC50/LEC values (Equations 1-3).

$$\text{Assay Score} = AS_i = -\log_{10}\left(\frac{AC50_i\, or LEC_i}{10^6}\right) \quad (1)$$

$$\text{Normalized Target Score} = \hat{T}S_i = \frac{\sum_j AS_i}{\left(\sum_j AS_i\right)_{\max}} \quad (2)$$

$$\text{Vascular Bioactivity Score} = VBS = \sum_i k_i \hat{T}S_i \quad (3)$$

Here, *i* represents the assay target (feature) and *j* represents the number of different assay systems each target was measured in. When applicable, the directional regulation of each of these targets with respect to blood vessel development was considered. For example, the upregulation of CXCL10, an anti-angiogenic chemokine, was considered to be relevant while downregulation was not, as suppression of that chemokine would provide an environment favorable for angiogenesis, whereas our hypothesis is focused on disruption of vascular processes. The assay scores were normalized and summed (Equation 2), and chemicals were ranked based on the Vascular Bioactivity Score (VBS) shown in Equation 3, where k_i is the weighted coefficient value determined by the number of term associations in the VT-KB search and by the specificity to vascular developmental processes: $k_{1,2}$=3 (VEGFR2, TIE2), $k_{3,4}$=2 (CCL2, PAI-1), and $k_{5,6}$=1 (CXCL10, uPAR). Those that had a VBS above the mean (123 chemicals out of 309) were classified as putative VDCs. The pVDC ToxPi profiles are shown in Supplemental Figure 2, where each sector is normalized to show the relative effect of each chemical on each read-out and the slice widths represent the relative weights (k_i) across VBS targets.

Multivariate Modeling

We used machine learning tools to build a step-wise linear discriminant analysis (LDA) algorithm that yields a multivariate toxicity signature based on significant associations between the features set (*in vitro* ToxCast assay data and pathway perturbation scores) and a pre-defined endpoint. Typically, the endpoints are *in vivo* phenotypes culled from guideline animal studies and reported in ToxRefDB. Vascular disruption may result in a variety of developmental endpoints including fetal resorption, limb defects and microphthalmia, and similarly these endpoints could be a result of disruption of other non-vascular developmental processes or maternal toxicity. In an attempt to separate out those compounds which were potentially acting via a vascular disruptive mechanism, we defined pVDCs as those chemicals which had a VBS

greater than the mean over the entire Phase 1 chemical space based solely on 6 assay targets critical to vascular development. We then identified those compounds with species-specific developmental toxicity and searched for correlations among the remainder of the ToxCast *in vitro* data and the aggregated pathway-perturbation scores.

First, individual (univariate) statistical associations were calculated for the chemical-assay and chemical-endpoint space. Two statistical tests were used. In the first, the data matrix was dichotomized so that if activity was seen at any concentration (assays) or dose (endpoints), a value of 1 was assigned to the chemical assay (endpoint) pair. Otherwise a value of 0 was assigned. Next, one assay was selected as the input (predictor variable) and another assay (or endpoint) as the output (predicted variable). A 2x2 contingency table was created with values for TP (true positive, number of chemicals for which the input and output were both positive), FP (false positive, number of chemicals for which the input was positive and output negative), FN (false negative, number of chemicals for which the input was negative and the output positive) and TN (true negative, both input and output negative). The significance of association was tested using a Fisher's exact test. In the second statistical method, the input assay AC50 values were log transformed and scaled as in Equation 1. This scaling yields a value of zero for inactive chemical-assay combinations. The output variable was dichotomized as before. We then performed a t-test comparing the score distribution for the output-positive vs. output-negative chemicals. Each pair of associations was ranked by the minimum p-value from either test, with a cut-off of $p<0.05$ designated as statistically significant.

Multivariate models were built where the species-specific pVDCs were designated as outputs and the ToxCast assays and pathway perturbation scores as inputs. The predictive model was constructed from the most significant univariate features, and cross-validated over

20 iterations. For this analysis, the original data matrix was log-transformed. The model is of the form,

$$\textit{Model Score (chemical}_x\textit{)} = Cutoff + \sum_{assay_i}^{N\max}\left[\delta_i AS_i\left(chemical_x\right)\right] \quad (4)$$

where if δ_i is 1, then assay *i* is included, otherwise it is not. If the model score for chemical *x* is >0, the chemical is predicted to be active in the output class or endpoint, otherwise it is predicted to be inactive. Assays are added to the sum in a stepwise fashion, where the one with the most significant univariate association is added first, the second most significant is added next and so on. Model performance was evaluated using a 2x2 contingency table as described above where the true activity vector for the output assay is compared with the predicted activity vector. The model was implemented using a k-fold cross validation algorithm in which the data is randomly divided into training (80%) and test (20%) portions and optimal linear combinations of features are found which maximize the area under the curve (AUC) of the Receiver Operator Characteristic (ROC) curve. In addition to the AUC and Fisher's exact p-value, we also calculated the sensitivity, specificity, balanced accuracy (average of sensitivity and specificity) and other metrics. The algorithm was run multiple times with varying feature sets, allowing for linear combinations of ToxCast *in vitro* assays (excluding the VBS ranking assays), ToxRefDB in *vivo* data, and pathway perturbation scores. The model with best cross-validation test balanced accuracy (BA, an average of sensitivity and specificity) was selected for further consideration. The algorithm is implemented in R and is available upon request ("linmod.R" (http://www.epa.gov/ncct/)).

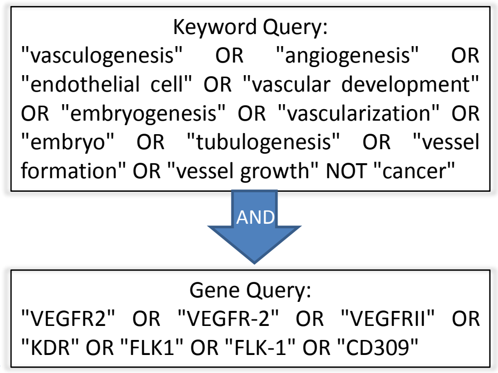

Gene	PubMed Abstracts
VEGFR-2	1775
Tie-2	937
PAI-1	743
CCL2	543
CXCL20	126
uPAR	118

Supplemental Material, Figure 1: Virtual Tissues Knowledge Base (VT-KB) example keyword query to rank ToxCast assay targets, where VEGFR-2 is used as an example. The 6 ToxCast^{TM} Phase I targets with high relevance to vascular development are shown.

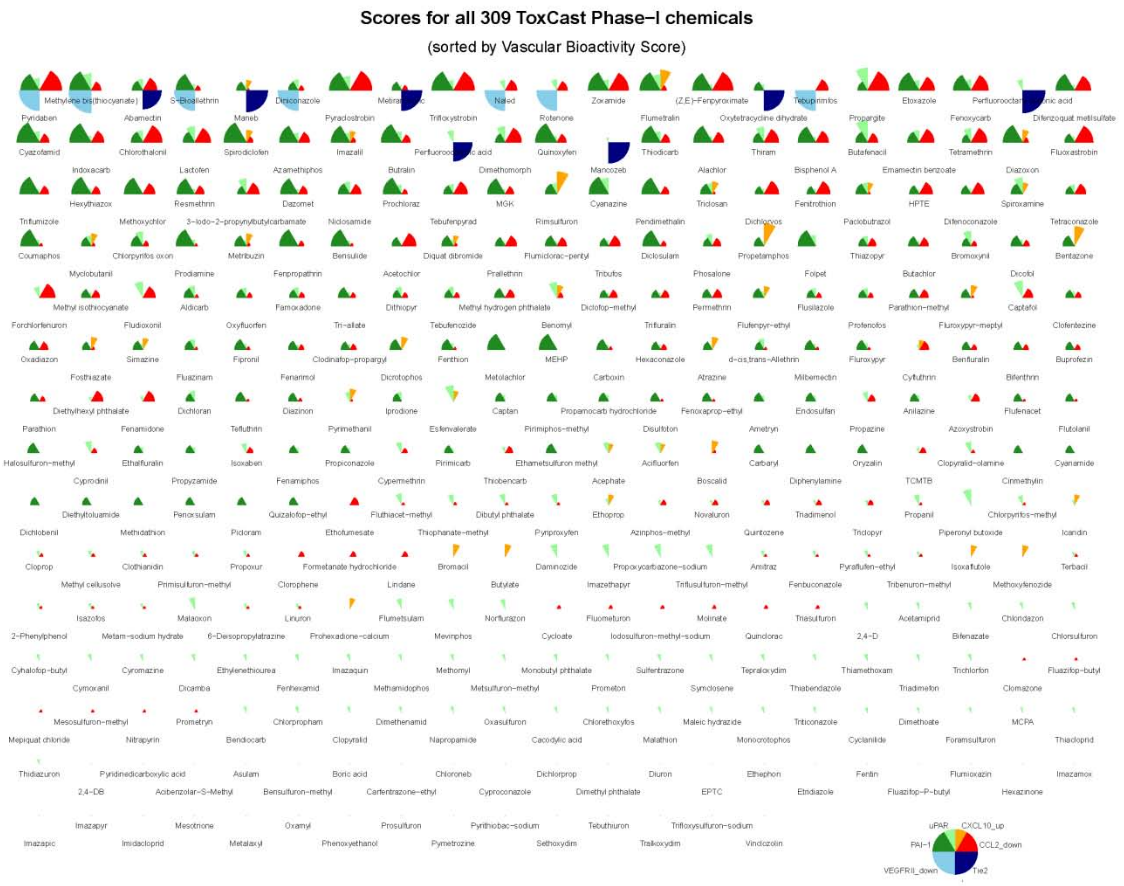

Supplemental Material, Figure 2: ToxPi visualization for all 309 ToxCast compounds ranked by VBS across 6 *in vitro* targets: CCL2↓, CXCL10↑, uPAR↕, PAI-1↕, VEGFR2↓, TIE2 binding. Arrows represent direction of regulation where applicable.

.

Chemical	RAT/RABBIT Developmental	RAT Chronic	MOUSE Chronic	RAT Multigenerational	VTKB Results (peer-reviewed in vivo studies)
Bentazone	No Study in ToxRefDB	AdrenalGland_AnyLesion, PreneoplasticLesion, NeoplasticLesion; Eye_AnyLesion; Lung_AnyLesion; Tumorigen; Uterus_AnyLesion, PreneoplasticLesion, NeoplasticLesion	Heart_AnyLesion; LiverProliferativeLesions; LiverTumors; Liver_AnyLesion, PreneoplasticLesion, NeoplasticLesion; Pancreas_AnyLesion, PreneoplasticLesion; Testes_AnyLesion; Tumorigen	Kidney; Liver	Oral Toxicity (Respiratory, Muscular): Pheasants, Rabbits (Neuschl and Kacmar 1993)
Coumaphos	No Study in ToxRefDB	CholinesteraseInhibition	No effect recorded	No effect recorded	No Evident Developmental Toxicity: Cows (Bellows et al. 1975); Delayed Neurotoxicity: Hens (Abou-Donia et al. 1982)
Forchlorfenuron	No Study in ToxRefDB	KidneyNephropathy; Kidney_AnyLesion; Ovary_AnyLesion; SpleenPathology; Spleen_AnyLesion; Thymus_AnyLesion; Thymus_PreneoplasticLesion	No Chronic Mouse Study in ToxRefDB	Kidney; LactationPND21; LitterSize; Liver; Ovary; Testis	No *in vivo* toxicity data returned
Milbemectin (mix of >70%Milbemycin A4; <30%Milbemycin A3)	No Study in ToxRefDB	KidneyNephropathy; Kidney_AnyLesion; Skin_AnyLesion	Heart_AnyLesion; KidneyPathology; Kidney_AnyLesion; LiverNecrosis; Liver_AnyLesion; Ovary_AnyLesion	No Study in ToxRefDB	No *in vivo* toxicity data returned
Prochloraz	No Study in ToxRefDB	Liver_AnyLesion	LiverProliferativeLesions; LiverTumors; Liver_AnyLesion, PreneoplasticLesion, NeoplasticLesion; Tumorigen	No Study in ToxRefDB	Reproductive Toxicity: Rats(Laier et al. 2006; Noriega et al. 2005; Vinggaard et al. 2005a; Wilson et al. 2004); Endocrine Disruptor: Rats(Vinggaard et al. 2005b), Zebrafish(Kinnberg et al. 2007), Trout(Le Gac et al. 2001); Behavioral Effects: Goldfish(Saglio et al. 2001)
Bromoxynil	No Study in ToxRefDB	Liver_AnyLesion; Thymus_AnyLesion	LiverHypertrophy; LiverNecrosis; LiverProliferativeLesions; LiverTumors; Liver_AnyLesion, PreneoplasticLesion, NeoplasticLesion; Tumorigen	No Study in ToxRefDB	Developmental Toxicity (Axial Skeletal): Rats, Mice(Kawanishi et al. 2003; Rogers et al. 1991)

Imazalil	No Study in ToxRefDB	LiverHypertrophy; LiverProliferativeLesions; LiverTumors; Liver_AnyLesion, PreneoplasticLesion, NeoplasticLesion; MammaryGland_AnyLesion; ThyroidGland_AnyLesion, PreneoplasticLesion, NeoplasticLesion; ThyroidHyperplasia; ThyroidProliferativeLesions; ThyroidTumors; Tumorigen	LiverHypertrophy; LiverProliferativeLesions; LiverTumors; Liver_AnyLesion, PreneoplasticLesion, NeoplasticLesion; Pancreas_AnyLesion; Tumorigen; Vagina_AnyLesion	Gestational Interval; Implantations; LactationPND21; LitterSize; LiveBirthPND1; Liver; ViabilityPND4	Neurobehavioral Effects: Mice(Tanaka 1995)
Niclosamide	No Study in ToxRefDB	No Study in ToxRefDB	No Study in ToxRefDB	No Study in ToxRefDB	Intestinal Parasite Treatment in Mammals: No Apparent Toxicity(Bryan 1976; Jones 1979)
Diazoxon	No Study in ToxRefDB	No Study in ToxRefDB	No Study in ToxRefDB	No Study in ToxRefDB	Chronic Health Effects: Humans (evidence of genetic suceptibility)(Mackness et al. 2003; Povey 2010)
Methoxychlor	No Study in ToxRefDB	No Study in ToxRefDB	No Study in ToxRefDB	No Study in ToxRefDB	Reproductive Toxicity: Mice, Rats(Cummings and Gray 1989; Cummings and Perreault 1990; Johnson et al. 1992), Monkeys(Tiemann 2008); Endocrine Disruptor: Mice, Rats, Zebrafish(Tiemann 2008), Quail(Ottinger et al. 2005)
2,2-Bis(4-hydroxyphenyl)-1,1,1-trichloroethane (HPTE)	No Study in ToxRefDB	No Study in ToxRefDB	No Study in ToxRefDB	No Study in ToxRefDB	Endocrine Disruptor: Mice, Rats(Murono and Derk 2005)
Chlorpyrifos oxon	No Study in ToxRefDB	No Study in ToxRefDB	No Study in ToxRefDB	No Study in ToxRefDB	Dvelopmental Toxicity: Rats(Chanda et al. 1995), Mice(Slotkin 1999); Neurotoxicity: Rats(Richardson 1995)
Oxytetracycline dihydrate	No Study in ToxRefDB	No Study in ToxRefDB	No Study in ToxRefDB	No Study in ToxRefDB	No *in vivo* toxicity data returned
Fluroxypyr-meptyl	No Study in ToxRefDB	No Study in ToxRefDB	No Study in ToxRefDB	No Study in ToxRefDB	Aquatic Toxicity(EFSA 2011)

Methyl hydrogen phthalate	No Study in ToxRefDB	No Study in ToxRefDB	No Study in ToxRefDB	No Study in ToxRefDB	No *in vivo* toxicity data returned
Perfluorooctanoic acid	No Study in ToxRefDB	No Study in ToxRefDB	No Study in ToxRefDB	No Study in ToxRefDB	Developmental Toxicity: Humans (low birth weight)(Fei et al. 2007), Rats(Lau et al. 2004), Mice(Lau et al. 2006; Wolf et al. 2007); Thyroid Disease: Humans(Melzer et al. 2010)
Abamectin	No Study in ToxRefDB	No Study in ToxRefDB	No Study in ToxRefDB	No Study in ToxRefDB	Neurotoxicity: Mice(Bloomquist 1992); Reproductive Toxicity: Rats(Elbetieha and Da'as 2003); Aquatic Toxicity: Trout(Jencic et al. 2006)

Supplemental Table 1: Predicted VDCs with no prenatal guideline study in ToxRefDB. VT-KB literature search results are reported.

Chemical	RAT/RABBIT Developmental	RAT Chronic	MOUSE Chronic	RAT Multigenerational	VTKB Results (peer-reviewed *in vivo* studies)
Zoxamide	No effect recorded	No effect recorded	LungTumors; Lung_ AnyLesion, PreneoplasticLesion, NeoplasticLesion; Tumorigen	No effect recorded	No *in vivo* toxicity data returned
Triclosan	No effect recorded	Blood_ AnyLesion; Kidney_ AnyLesion; LiverHypertrophy; LiverNecrosis; Liver_ AnyLesion; Lung_ AnyLesion	No Chronic Mouse Study in ToxRefDB	Kidney; ViabilityPND4	Developmental toxicity: Zebrafish(Oliveira et al. 2009); Liver/Kidney toxicity: Rats, Hamsters(Rodricks et al. 2010); Endocrine Disruptor: Rats(Kumar et al. 2009), Sheep Placenta(James et al. 2010)
MGK	No effect recorded	AdrenalGland_ AnyLesion, PreneoplasticLesion; BoneMarrow_ AnyLesion, PreneoplasticLesion; Bone_ AnyLesion; Eye_ AnyLesion; Kidney_ AnyLesion; LiverHypertrophy; LiverProliferativeLesions; Liver_ AnyLesion, PreneoplasticLesion; Lung_ AnyLesion; LymphNode_ AnyLesion; Ovary_ AnyLesion; Skin_ AnyLesion, PreneoplasticLesion, NeoplasticLesion; Stomach_ AnyLesion; Testes_ AnyLesion; ThyroidGland_ AnyLesion, PreneoplasticLesion, NeoplasticLesion; ThyroidHyperplasia; ThyroidProliferativeLesions; ThyroidTumors; Tumorigen; UrinaryBladder_ AnyLesion, PreneoplasticLesion	Gallbladder_ AnyLesion; Heart_ AnyLesion; KidneyPathology; Kidney_ AnyLesion; LiverHypertrophy; LiverProliferativeLesions; LiverTumors; Liver_AnyLesion, PreneoplasticLesion, NeoplasticLesion; Tumorigen	Liver	No *in vivo* toxicity data returned
Dithiopyr	No effect recorded	KidneyNephropathy; Kidney_ AnyLesion; LiverNecrosis; LiverProliferativeLesions; Liver_ AnyLesion, PreneoplasticLesion	AdrenalGland_ AnyLesion; KidneyPathology; Kidney_ AnyLesion, PreneoplasticLesion; LiverHypertrophy; LiverProliferativeLesions; Liver_ AnyLesion, PreneoplasticLesion	Adrenal; Kidney; Liver; Thyroid	No *in vivo* toxicity data returned

Tebufenozide	No effect recorded	Brain_ AnyLesion; Liver_ AnyLesion; PituitaryGland_ AnyLesion, PreneoplasticLesion, NeoplasticLesion; SpleenPathology; Spleen_ AnyLesion; Tumorigen	Spleen_ AnyLesion	Ovary; Spleen; Testis; Uterus	Immunomodulator: Lake Trout(Hamoutene et al. 2008)
Fosthiazate	No effect recorded	AdrenalGland_ AnyLesion; CholinesteraseInhibition; Eye_ AnyLesion; Liver_ AnyLesion; Lung_ AnyLesion; Ovary_ AnyLesion; PituitaryGland_ AnyLesion; SkeletalMuscle_ AnyLesion	AdrenalGland_ AnyLesion; KidneyPathology; Kidney_ AnyLesion; PituitaryGland_ AnyLesion	Adrenal; LactationPND21; LiveBirthPND1; Liver; ViabilityPND4	High Toxicity Risk/Environmental Persistence: Humans(Sanchez-Moreno et al. 2009)
Fipronil	No effect recorded	KidneyNephropathy; Kidney_ AnyLesion; ThyroidGland_ AnyLesion, PreneoplasticLesion, NeoplasticLesion; ThyroidProliferativeLesions; ThyroidTumors; Tumorigen	LiverHypertrophy; LiverNecrosis; LiverProliferativeLesions; Liver_ AnyLesion, PreneoplasticLesion	Epididymis; Fertility; LitterSize; LiveBirthPND1; Liver; Mating; Ovary; Thyroid; ViabilityPND4	Developmental Neurotoxicity: Zebrafish(Stehr et al. 2006)

Supplemental Table 2: Predicted VDCs with no prenatal effect recorded in ToxRefDB. VT-KB literature search results are reported.

Supplemental References:

Abou-Donia MB, Makkawy HA, Graham DG. 1982. Coumaphos: delayed neurotoxic effect following dermal administration in hens. J Toxicol Environ Health 10(1):87-99.
Bellows RA, Rumsey TS, Kasson CW, Bond J, Warwick EJ, Pahnish OF. 1975. Effects of organic phosphate systemic insecticides on bovine embryonic survival and development. Am J Vet Res 36(08):1133-1140.
Bloomquist JR. 1992. Intrinsic lethality of chloride-channel-directed insecticides and convulsants in mammals. Toxicol Lett 60(3):289-298.
Bryan RP. 1976. Anthelmintic treatment of young beef cattle in the Wallum region of south-eastern Queensland. Aust Vet J 52(9):403-408.
Chanda SM, Harp P, Liu J, Pope CN. 1995. Comparative developmental and maternal neurotoxicity following acute gestational exposure to chlorpyrifos in rats. J Toxicol Environ Health 44(2):189-202.
Cummings AM, Gray LE, Jr. 1989. Antifertility effect of methoxychlor in female rats: dose- and time-dependent blockade of pregnancy. Toxicol Appl Pharmacol 97(3):454-462.
Cummings AM, Perreault SD. 1990. Methoxychlor accelerates embryo transport through the rat reproductive tract. Toxicol Appl Pharmacol 102(1):110-116.
EFSA EFSA. 2011. Conclusion on the peer review of the pesticide risk assessment of the active substance fluroxypyr (evaluated variant fluroxypyr-meptyl). EFSA Journal 9(3):2091.
Elbetieha A, Da'as SI. 2003. Assessment of antifertility activities of abamectin pesticide in male rats. Ecotoxicol Environ Saf 55(3):307-313.
Fei C, McLaughlin JK, Tarone RE, Olsen J. 2007. Perfluorinated chemicals and fetal growth: a study within the Danish National Birth Cohort. Environ Health Perspect 115(11):1677-1682.
Hamoutene D, Payne JF, Volkoff H. 2008. Effects of tebufenozide on some aspects of lake trout (Salvelinus namaycush) immune response. Ecotoxicol Environ Saf 69(2):173-179.
James MO, Li W, Summerlot DP, Rowland-Faux L, Wood CE. 2010. Triclosan is a potent inhibitor of estradiol and estrone sulfonation in sheep placenta. Environ Int 36(8):942-949.
Jencic V, Cerne M, Erzen NK, Kobal S, Cerkvenik-Flajs V. 2006. Abamectin effects on rainbow trout (Oncorhynchus mykiss). Ecotoxicology 15(3):249-257.
Johnson DC, Sen M, Dey SK. 1992. Differential effects of dichlorodiphenyltrichloroethane analogs, chlordecone, and 2,3,7,8-tetrachlorodibenzo-p-dioxin on establishment of pregnancy in the hypophysectomized rat. Proc Soc Exp Biol Med 199(1):42-48.
Jones WE. 1979. Niclosamide as a treatment for Hymenolepis diminuta and Dipylidium caninum infection in man. Am J Trop Med Hyg 28(2):300-302.
Kawanishi CY, Hartig P, Bobseine KL, Schmid J, Cardon M, Massenburg G, et al. 2003. Axial skeletal and Hox expression domain alterations induced by retinoic acid, valproic acid, and bromoxynil during murine development. J Biochem Mol Toxicol 17(6):346-356.
Kinnberg K, Holbech H, Petersen GI, Bjerregaard P. 2007. Effects of the fungicide prochloraz on the sexual development of zebrafish (Danio rerio). Comp Biochem Physiol C Toxicol Pharmacol 145(2):165-170.

Kumar V, Chakraborty A, Kural MR, Roy P. 2009. Alteration of testicular steroidogenesis and histopathology of reproductive system in male rats treated with triclosan. Reprod Toxicol 27(2):177-185.
Laier P, Metzdorff SB, Borch J, Hagen ML, Hass U, Christiansen S, et al. 2006. Mechanisms of action underlying the antiandrogenic effects of the fungicide prochloraz. Toxicol Appl Pharmacol 213(2):160-171.
Lau C, Butenhoff JL, Rogers JM. 2004. The developmental toxicity of perfluoroalkyl acids and their derivatives. Toxicol Appl Pharmacol 198(2):231-241.
Lau C, Thibodeaux JR, Hanson RG, Narotsky MG, Rogers JM, Lindstrom AB, et al. 2006. Effects of perfluorooctanoic acid exposure during pregnancy in the mouse. Toxicol Sci 90(2):510-518.
Le Gac F, Thomas JL, Mourot B, Loir M. 2001. In vivo and in vitro effects of prochloraz and nonylphenol ethoxylates on trout spermatogenesis. Aquat Toxicol 53(3-4):187-200.
Mackness B, Durrington P, Povey A, Thomson S, Dippnall M, Mackness M, et al. 2003. Paraoxonase and susceptibility to organophosphorus poisoning in farmers dipping sheep. Pharmacogenetics 13(2):81-88.
Melzer D, Rice N, Depledge MH, Henley WE, Galloway TS. 2010. Association between serum perfluorooctanoic acid (PFOA) and thyroid disease in the U.S. National Health and Nutrition Examination Survey. Environ Health Perspect 118(5):686-692.
Murono EP, Derk RC. 2005. The reported active metabolite of methoxychlor, 2,2-bis(p-hydroxyphenyl)-1,1,1-trichloroethane, inhibits testosterone formation by cultured Leydig cells from neonatal rats. Reprod Toxicol 20(4):503-513.
Neuschl J, Kacmar P. 1993. [Acute oral toxicity of bentazon, an herbicide developed in Czechoslovakia, in pheasants and rabbits and the clinical symptoms of poisoning]. Vet Med (Praha) 38(2):115-121.
Noriega NC, Ostby J, Lambright C, Wilson VS, Gray LE, Jr. 2005. Late gestational exposure to the fungicide prochloraz delays the onset of parturition and causes reproductive malformations in male but not female rat offspring. Biol Reprod 72(6):1324-1335.
Oliveira R, Domingues I, Koppe Grisolia C, Soares AM. 2009. Effects of triclosan on zebrafish early-life stages and adults. Environ Sci Pollut Res Int 16(6):679-688.
Ottinger MA, Quinn MJ, Jr., Lavoie E, Abdelnabi MA, Thompson N, Hazelton JL, et al. 2005. Consequences of endocrine disrupting chemicals on reproductive endocrine function in birds: establishing reliable end points of exposure. Domest Anim Endocrinol 29(2):411-419.
Povey AC. 2010. Gene-environmental interactions and organophosphate toxicity. Toxicology.
Richardson RJ. 1995. Assessment of the neurotoxic potential of chlorpyrifos relative to other organophosphorus compounds: a critical review of the literature. J Toxicol Environ Health 44(2):135-165.
Rodricks JV, Swenberg JA, Borzelleca JF, Maronpot RR, Shipp AM. 2010. Triclosan: a critical review of the experimental data and development of margins of safety for consumer products. Crit Rev Toxicol 40(5):422-484.
Rogers JM, Francis BM, Barbee BD, Chernoff N. 1991. Developmental toxicity of bromoxynil in mice and rats. Fundam Appl Toxicol 17(3):442-447.
Saglio P, Olsen KH, Bretaud S. 2001. Behavioral and olfactory responses to prochloraz, bentazone, and nicosulfuron-contaminated flows in goldfish. Arch Environ Contam Toxicol 41(2):192-200.

Sanchez-Moreno S, Alonso-Prados E, Alonso-Prados JL, Garcia-Baudin JM. 2009. Multivariate analysis of toxicological and environmental properties of soil nematicides. Pest Manag Sci 65(1):82-92.
Slotkin TA. 1999. Developmental cholinotoxicants: nicotine and chlorpyrifos. Environ Health Perspect 107 Suppl 1:71-80.
Stehr CM, Linbo TL, Incardona JP, Scholz NL. 2006. The developmental neurotoxicity of fipronil: notochord degeneration and locomotor defects in zebrafish embryos and larvae. Toxicol Sci 92(1):270-278.
Tanaka T. 1995. Reproductive and neurobehavioral effects of imazalil administered to mice. Reprod Toxicol 9(3):281-288.
Tiemann U. 2008. In vivo and in vitro effects of the organochlorine pesticides DDT, TCPM, methoxychlor, and lindane on the female reproductive tract of mammals: a review. Reprod Toxicol 25(3):316-326.
Vinggaard AM, Christiansen S, Laier P, Poulsen ME, Breinholt V, Jarfelt K, et al. 2005a. Perinatal exposure to the fungicide prochloraz feminizes the male rat offspring. Toxicol Sci 85(2):886-897.
Vinggaard AM, Jacobsen H, Metzdorff SB, Andersen HR, Nellemann C. 2005b. Antiandrogenic effects in short-term in vivo studies of the fungicide fenarimol. Toxicology 207(1):21-34.
Wilson VS, Lambright C, Furr J, Ostby J, Wood C, Held G, et al. 2004. Phthalate ester-induced gubernacular lesions are associated with reduced insl3 gene expression in the fetal rat testis. Toxicol Lett 146(3):207-215.
Wolf CJ, Fenton SE, Schmid JE, Calafat AM, Kuklenyik Z, Bryant XA, et al. 2007. Developmental toxicity of perfluorooctanoic acid in the CD-1 mouse after cross-foster and restricted gestational exposures. Toxicol Sci 95(2):462-473.
